# Simultaneous determination of brazilin and protosappanin B in *Caesalpinia sappan* by ionic-liquid dispersive liquid-phase microextraction method combined with HPLC

**DOI:** 10.1186/s13065-017-0342-9

**Published:** 2017-11-13

**Authors:** Zhaoyang Xia, Dongdong Li, Qing Li, Yan Zhang, Wenyi Kang

**Affiliations:** 1 Institute of Chinese Materia Medica, Henan University, Kaifeng, Henan 475004 China; 2 Kaifeng Key Laboratory of Functional Components in Health Food, Kaifeng, Henan 475004 China; 3 Hebei Food Inspection and Research Institute, Shijiazhuang, 050091 China

**Keywords:** *Caesalpinia sappan*, Brazilin, Protosappanin B, Ionic liquids, HPLC

## Abstract

The conditions of heating, ionic liquid-based ultrasonic-assisted extraction combined with reverse-phase high performance liquid chromatography were optimized to simultaneously isolate and determinate brazilin and protosappanin B in *Caesalpinia sappan*. Ionic liquids, including [BMIM]Br, [BMIM]BF_4_, [BMIM]PF_6_ and [HMIM]PF_6_, were selected as extraction solvents while methanol, acetone, acetonitrile, ethanol and water were selected as dispersants. The chromatographic column was Purospher star RP-C_18_ (250 mm × 4.6 mm, 5 μm), a mixture of methanol and 0.2% phosphoric acid–water was used as mobile phase at a flow rate 0.65 mL/min. The result displayed that the extraction yields of brazilin and protosappanin B were highest when the concentration of [BMIM]Br methanol solution as extraction solvent was 0.5 mol/L and the solid–liquid ratio was 1:50 (g/mL). Under the optimal extraction conditions, the contents of brazilin showed a good linearity (*r* = 1.0000) within the range of 1.25–7.50 μg with the average recovery of 99.33%, the contents of protosappanin B also showed a good linearity (*r* = 0.9999) within the range of 0.50–3.00 μg with the average recovery of 98.31%. This experiment, which adopted environmentally friendly reagent as extraction solvent, not only improved the extraction efficiency, but also avoided the environmental pollution caused by organic solvent. Moreover, it was simple and reliable, and can be of important significance in the study of Traditional Chinese Medicine active ingredient extraction methods. The antibacterial activities of the ionic liquids and methanol extracts were determined using the paper disc diffusion method. The ionic liquid extract was found to possess antibacterial activity against *Staphylococcus aureus* and methicillin-resistant *S. aureus* (MIC value of 37.5 mg crude drug/mL), *β*-Lactamase producing *S. aureus* (MIC values of 18.8 mg crude drug/mL), but not against *E. coli*, Extended spectrum *β*-Lactamases *E. coli* and *P. aeruginosa*. Compared with the ionic liquid extract, the methanol extract was found to have antibacterial activity against *S. aureus* and methicillin-resistant *S. aureus* (MIC value of 75.0 mg crude drug/mL), *β*-Lactamase producing *S. aureus* (MIC values of 150.0 mg crude drug/mL). However, the same, the methanol extract did not have antibacterial activity against *E. coli*, Extended spectrum *β*-Lactamases *E. coli* and *P. aeruginosa*.

## Introduction

*Caesalpinia sappan* L. (*C. sappan*) belonging to family Caesalpiniaceae, an important source for pink and bright reddish-purple colors, has been cultivated in South-East Asia for the production of red dye, which is obtained from its heartwood. The heartwood of this plant has long been used as blood tonic, emmenagogue, and expectorant [1]. Pharmacological researches have shown that *C. sappan* possesses a wide range of pharmacological activities, including antibacterial [2, 3], antioxidant [4, 5], anti-inflammatory [6, 7], anticarcinogenic [8, 9] and apoptosis-inducing activities in Hela cells [10].

Brazilin and protosappanin B are two major bioactive homoisoflavones isolated from *C. sappan*, which are used as the marker components for the quality control of the crude drug in the current version of China Pharmacopoeia [11]. Lee et al. [12] found that brazilin exhibited anti-T_H_2 effects both in vitro and in vivo. Tong et al. [13] found that elimination rates of brazilin and protosappanin B in the streptozotocin-treated rats were slower when compared with those of the normal rats, suggesting that the accumulation of both compounds in diabetic pathologic states and their adverse reactions should be considered. Zeng et al. [14] found that protosappanin B significantly increased cell viability, inhibited cell apoptosis and up-regulated the expression of growth-associated protein 43. In order to investigate their pharmacological and toxicological properties, we need the methods for rapid, accurate and simultaneous determination of both brazilin and protosappanin B. However, to date, few high-performance liquid chromatography (HPLC) methods for the simultaneous determination of both brazilin and protosappanin B in the crude drug have been reported in the literature [15–17], and also have many shortcomings, for example, low yield, many impurities and poor quality.

Because of their low concentrations, it is very important to improve the limit of detection of these analytes and to achieve good results. To improve the extraction rate, highly toxic solvents are often used. However, the use of toxic solvents can cause pollution to the environment.

In the recent years, more and more researchers have paid increasing attention to a new type of green solvent-ionic liquids. Ionic liquids have many advantages, such as lower melting point, less volatility, higher solubility, chemical stabilization and other characteristics and can be designed to regulate [18]. Ionic liquids have been an alternative to volatile, toxic and hazardous organic solvents in many areas of organic synthesis, electrochemistry, and catalysis [19]. When being used as the extraction solvent in the progress of plant, it can destroy the structure of cellulose, and dissolve cellulose with ionic liquids, ultimately, promoting the dissolution of effective ingredients in the solution [20]. It is not only environmentally friendly, but also can greatly increase the extraction rate of compounds.

In addition to ionic liquids as extractants, the different solvents were also used as disperser solvents to improve the content of brazilin and protosappanin B in *C. sappan*. Disperser solvents should be able to completely dissolved extraction solvent. The effect of dispersant is to achieve maximum contact area between the extraction solvent and sample. The greater the solubility in water of the dispersant is, the smaller the formation of droplet, and the greater the contact area of the target, and the higher extraction efficiency are.

Recently, ultrasound and temperature [21] have been used to improve the extraction rate. Ultrasound can destroy the cellular structure, leading to the significant increase of mass transfer of analytes into the extractant. Increased extraction temperature is a force driving dispersion of the extraction solvent into an aqueous solution, which can also increase the contact area between the ionic liquids and sample solution and thus, affect the rates of mass transfer of the analytes [22].

According to Ref. [23], this study aimed to evaluate the performance of extraction solvent, disperser solvents, extractant concentration, solid–liquid ratio, ultrasound, temperature and centrifugal rotational speed on the extraction efficiency and to finally find out the optimal extraction conditions, so that the extraction efficiency can be greatly increased.

## Materials and methods

### Plant, reagents and solutions

Heartwood (*Caesalpinia sappan*)(*C. sappan*) were collected from Yunnan, China, in July 2012, and identified by a plant scientist, Professor Changqin Li of Henan University. A voucher specimen (No. 20120710) was deposited in the herbarium of Institute of Natural Products, Henan University. The samples were crushed up and dried at room temperature. The air-dried sample was ground into fine powder and passed through a stainless steel sieve. Samples with different sizes were obtained and stored at room temperature in desiccator until use.

1-butyl-3-methylimidazolium hexafluorophosphate ([BMIM]PF_6_), 1-butyl-3- methylimidazolium bromide ([BMIM]Br) and 1-butyl-3-methylimidazolium tetrafluoroborate ([BMIM]BF_4_) were purchased from Merck KGaA (Germany); 1-hexyl-3-methylimidazolium hexafluorophosphate (HMIM]PF_6_) was purchased from Tokyo Chemical industry Co., Ltd.(Tokyo, Japan). Methanol obtained from Avantor Performance Materials, Inc. (Center Valley, PA, USA) was chromatographic grade. Phosphoric acid obtained from Tianjin Fu Chen Chemical Reagent Co., Ltd. (Tianjin, China) was of analytical grade.

Brazilin and protosappanin B as standards were purchased from the Chendu Preferred Biotechnology Co., Ltd. (Chengdu, Sichuan, China).

A mixed stock solution containing brazilin at 0.625 g/L and protosappanin B at 0.25 g/L was prepared in HPLC-grade methanol. Standard solutions were prepared by mixing appropriate amounts of the stock solution with ultra-pure water in 5 mL volumetric flasks. All the standard solutions were stored at 4 °C.

### Apparatus

The HPLC instrumentation equipped with a vacuum degasser, a quaternary pump (LC-20AD, SHIMADZU), a column oven (CTO-20AC, SHIMADZU), and an auto-sampler (SIL-20AC, SHIMADZU), was connected to a SPD-20AV UV–Vis detector (SHIMADZU). Chromatographic analysis was performed on a Purospher star RP-C_18_ column (4.6 mm × 250 mm, 5 μm) by using a simultaneous elution and flow-rate programming RP-HPLC method.

### Bacterial strains

*Staphylococcus aureus* (SA), methcillin-resistant *Staphylococcus aureus* (MRSA), *β*-lactamase-producing *Staphylococcus aure*us, *Escherichia coli* (EC), extended spectrum *β*-lactamases *Escherichia coli* (ESBL_S_-EC) and *Pseudomonas aeruginosa* (PA) were used as reference strains, following the guidelines of the Clinical and Laboratory Standards Institute. All the strains were the clinical isolates of bacterial species of interest selected from Huaihe Hospital, Henan University, China, VITEK-AMS identification, the purity greater than 99%.

### Chromatographic conditions

**Fig. 1:**
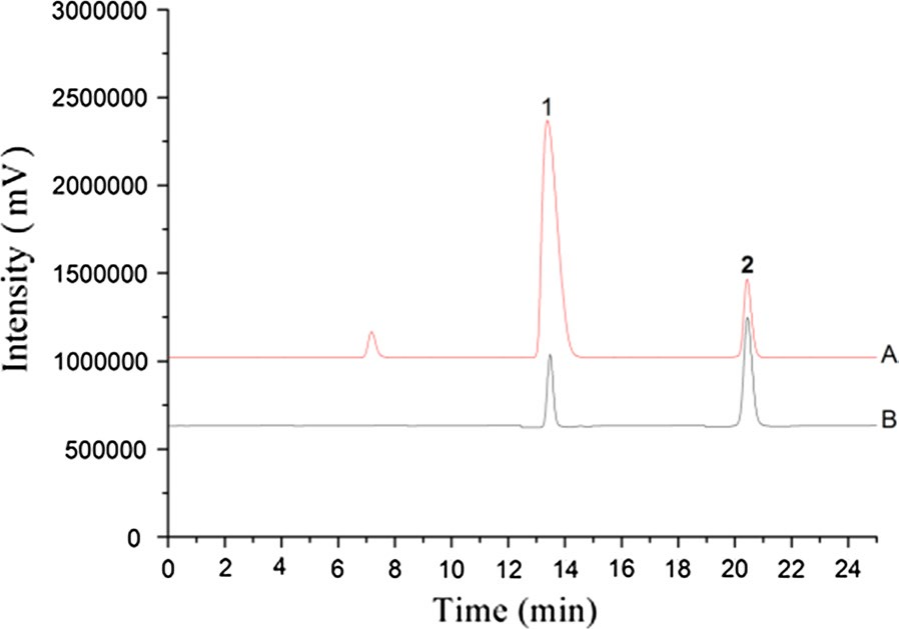
HPLC chromatograms of reference substance and test solution. A, Test solution of *Caesalpinia sappan*; B, mixed standard substance; Peak 1, brazilin; Peak 2, protosappanin B

The mobile phase was consisted of methanol–0.2% phosphoric acid. The gradient elution steps were set as follows: 0–25 min, 30% methanol, 70% phosphoric acid at a flow rate of 0.65 mL/min. The UV detection wavelength was 286 nm. Results were shown in Fig. 1.

### The influencing factors of extraction efficiency

#### Selection of type of ionic liquid extraction solvent

Two kinds of hydrophilic ([BMIM]Br and [BMIM]BF_4_) and two kinds of hydrophobic ([BMIM]PF_6_ and [HMIM]PF_6_) ionic liquids were used as the extraction solvents to research the rapid analysis method for ionic liquid microextraction on *C. sappan*, to compare the extraction efficiency of the target analytes and to select a suitable type of ionic liquid extraction solvent, respectively.

100 mg of *C. sappan* powder (50 mesh) was accurately weighed with five samples, and placed in a centrifuge tube with plug, add 5.0 mL of [BMIM]Br, [BMIM]BF_4_, [BMIM]PF_6_ and [HMIM]PF_6_ methanol solution whose concentrations were both 0.5 mol/L precisely, 5.0 mL of methanol in the control group, ultrasonic processing for 30 min at 35 °C. The solutions were centrifuged at 5000 r/min for 5 min. Solution was filtrated through 0.22 μm organic membrane, and the subsequent filtrates were collected. All the samples were automatically injected, and the peak area of each component was recorded.

#### Selection of type of non-ionic liquid disperser solvents

Five kinds of dispersants (methanol, water, acetone, acetonitrile and ethanol), which could be dissolved [BMIM]Br, were investigated on the extraction efficiency of brazilin and protosappanin B in *C. sappan*. These dispersants were used as dispersant. The final concentration of the extract was made at 0.5 mol/L and the extraction rates of the target analytes were compared, respectively.

100 mg of *C. sappan* powder (50 mesh) was accurately weighed with five samples, and 5.0 mL of methanol, water, acetone, acetonitrile and ethanol were added precisely with ultrasonic processing for 30 min at 35 °C, and then centrifuged for 5 min at 5000 r/min for 5 min.

#### Selection of extract concentration

The influences of [BMIM]Br methanol at concentrations of 0.1, 0.2, 0.3, 0.4, 0.5, 0.6, 0.7, 0.8 and 0.9 mol/L on the extraction rate were investigated, respectively. 100 mg of *C. sappan* powder (50 mesh sieve) was accurately weighed for nine samples, 5.0 mL of [BMIM]Br methanol solution was added, precisely, and with ultrasonic processing for 30 min at 35 °C, and then centrifuged for 5 min at 5000 r/min for 5 min.

#### Selection of mesh

Mesh of crushed herbs have some influences on the extraction rate, the smaller the powder is, the higher the extraction rate is. However, powder is easy to gather into ball when it is very small. Thus, the extraction rate would be affected by the powder size.

The effects of 10, 24, 40, 50, 70 and 90 mesh sieve powders the extraction efficiency of target analytes were assayed. 100 mg of *C. sappan* powder was accurately weighed for nine samples, 5.0 mL of [BMIM]Br methanol solution was adjusted to the concentration of 0.5 mol/L precisely with ultrasonic processing 30 min at of 35 °C, and centrifuged at 5000 r/min for 5 min.

#### Selection of solid–liquid ratio

The solid–liquid ratios at 1:20, 1:30, 1:50, 1:75, 1:100 and 1:125 (g/mL) were selected to investigate their effects on extract fluence of brazilin and protosappanin B in *C. sappan*. The *C. sappan* powder (70 mesh sieve) was accurately weighed for six different samples, 5.0 mL of [BMIM]Br methanol solution at concentration of 0.5 mol/L was added precisely with ultrasonic processing 30 min at 35 °C, and then centrifuged at 5000 r/min for 5 min.

#### Selection of ultrasonic time

Generally, the longer the ultrasonic time is, the higher extraction efficiency is. However, a long ultrasonic time can destroy the structure of the target compound. In this experiment, ultrasonic times for 10, 20, 30, 40, 50 and 60 min were examined for their effects on the extract efficiency of brazilin and protosappanin B in *C. sappan*.

100 mg of *C. sappan* powder (70 mesh sieve) in six samples were adjusted to the concentration at 0.5 mol/L with [BMIM]Br methanol solution precisely, and solid–liquid ratio was formulated into 1:50 sample and treated with ultrasonic extraction times for 10, 20, 30, 40, 50 and 60 min at 35, and centrifuged at 5000 r/min for 5 min.

#### Selection of ultrasonic temperature

Because of the boiling point of ionic-liquid at 75 °C, the highest temperature of ultrasonic temperature and heating temperature were below the boiling point. Concentration of *C. sappan* powder (70 mesh sieve) in [BMIM]Br methanol solution was 0.5 mol/L for five samples with ultrasonic extraction 50 min at 30, 40, 50, 60, and 70 °C, respectively, The solutions were centrifuged at 5000 r/min for 5 min.

#### Selection of heating time

Six samples of *C. sappan* powder (70 mesh sieve) in [BMIM]Br methanol solution at the concentration of 0.5 mol/L were heated for 10, 20, 30, 40, 50 and 60 min at 50 °C, and the solutions were centrifuged for 5 min at 5000 r/min for 5 min.

#### Selection of heating temperature

Five samples of *C. sappan* powder (70 mesh sieve) in [BMIM]Br methanol solution at the concentration of 0.5 mol/L were heated for 50 min at 30, 40, 50, 60, and 70 °C for 50 min The solutions were then centrifuged for 5 min at 5000 r/min for 5 min.

#### Selection of extraction method

Through the above tests, the optimum conditions for ultrasonic extraction were determined as follows: disperser solvent, the [BMIM]Br methanol solution; the concentration of *C. sappan* powder (70 mesh sieve), 0.5 mol/L; solid–liquid ratio, 1:50 sample; ultrasonic extraction time, 50 min at 50 °C, and centrifuge for 5 min at 5000 r/min. The optimum method for heat extraction as follows: the concentration of [BMIM]Br methanol solution, both 0.5 mol/L which was added precisely to *C. sappan* powder (70 mesh sieve); a solid–liquid ratio, 1:50 sample; extraction time, 50 min; extraction temperature; 60 °C, and centrifuge for 5 min at 5000 r/min. The optimum method for reflux extraction as follows: [BMIM]Br methanol solution at concentration of 0.5 mol/L was added precisely to *C. sappan* powder (70 mesh sieve), a solid–liquid ratio, 1:50 sample with reflux; extraction time, 50 min; and centrifuged at 5000 r/min for 5 min.

#### Selection of centrifugal rotational speed

In this experiment, centrifugal rotational speeds at 3000, 4000, 5000, 6000, 7000 and 8000 r/min were investigated to examine the influences of the centrifugal rotational speed on the experimental results.

The [BMIM]Br methanol solution at concentration of 0.5 mol/L , was precisely added to* C. sappan* powder (70 mesh sieve) in a solid–liquid ratio of 1:50. The sample was extracted with ultrasonic extraction for 50 min at 50 °C, and centrifuged for at different rotational speeds for 5 min.

### Antibacterial activities of different extracts

#### Preparation of the tested substance solutions

According to the optimal extraction conditions, *C. sappan* powder was extracted and collected for the subsequent filtrate, the filtrate decompression was enriched in vacuo, and diluted with [BMIM]Br methanol solution whose concentration was 0.5 mol/L to 0.2 mol/L. Methanol extract was the same as the above.

#### Paper disc diffusion method

The initial concentration of the tested substance solutions was 300 mg crude drug/mL. Sterile paper discs were loaded with 8 μL of solution and dried in a sterile room. At the same time, sterile paper discs were loaded with 8 μL of brazilin solution or protosappanin B solution, whose concentrations were both 10 mg/mL. Streptomycin was used as positive control and paper discs treated with methanol or [BMIM]Br methanol solution were used as the negative control. The paper disc diffusion method was used to determine antibacterial activity based on the method described by Ali et al. [24]. The plates were then incubated at 37 °C for 18 h in a water-jacket thermostatic incubator. Inhibition zone diameters (IZ) of the disc were measured and recorded at the end of the incubation time [25].

#### Determination of minimum inhibitory concentration (MIC)

The tested samples were dissolved in [BMIM]Br methanol solution and in methanol for brazilin, protosappanin B and streptomycin. Twofold serial dilutions of each compound were prepared [26]. According to the operation described above, the plates were then incubated at 37 °C for 18 h in the incubator. The MIC was defined as the lowest concentration of tested substance solutions, brazilin, protosappanin B and streptomycin at which no visible growth was observed. All the operations were performed under sterile conditions [27].

## Results and discussion

### Linear relationship study

According to the chromatographic condition and the sample processing method, a series of the mixed stock solutions with the injection volumes of 2, 4, 6, 8, 10 and 12 μL were analyzed by HPLC. The injection quality was taken as abscissa (X, μg), peak area value was taken as ordinate (Y), to linear fit. Brazilin regression equation is Y = 1653011.383X + 75841, *r* = 1.0000, and the linear range is 1.25–7.50 μg. Protosappanin B regression equation is Y = 1186313.371X + 15359, *r* = 1.0000, the linear range is 0.5–3.0 μg.

### The influencing factors of extraction efficiency

#### Selection of type of ionic liquid extraction solvent

**Fig. 2:**
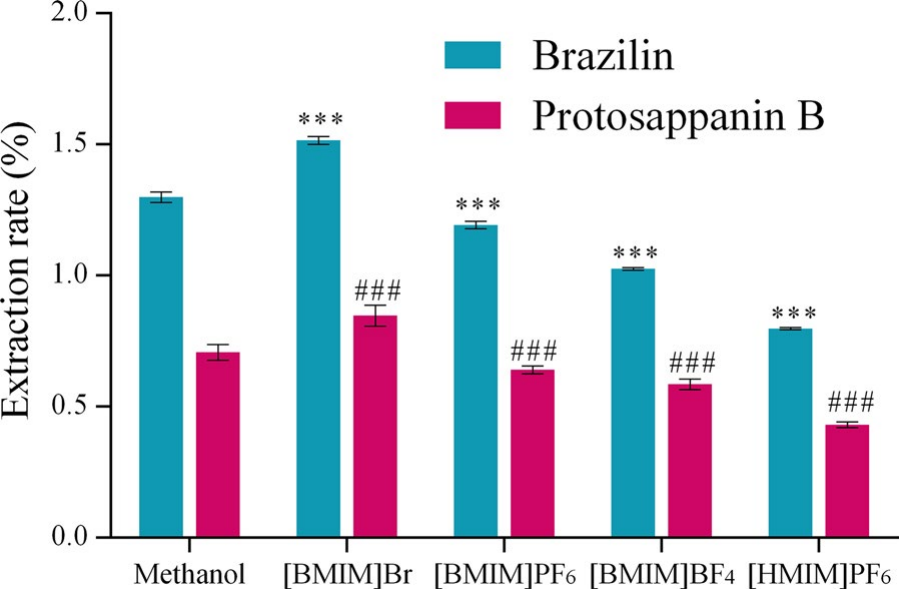
Influences of ionic liquids on the extraction rate of the compound. Note: compared with methanol (***P ≤ 0.001 or ^###^ *P* ≤ 0.001)

The comparison in extraction efficiency between ionic liquid and methanol was shown in Fig. 2. When [BMIM]Br methanol solution was used as extraction solvent, extraction rates of brazilin and protosappanin B were significantly higher than that of methanol, which were 1.52 and 0.85%, respectively. While the extraction rates of the other three kinds of ionic liquids were lower than that of methanol. The extraction rate of the [HMIM]PF_6_ was the lowest one, which were 0.80 and 0.43%, respectively. The cation of [BMIM]Br, [BMIM]BF_4_ and [BMIM]PF_6_ was 1-butyl-3-methylimidazolium, the solubility of ionic liquid depended on its anion. The solubility of ionic liquids directly affected the extraction rate of the target compound. At the same time, the damages of ionic liquids with different pH values to medicinal cells were also different, which immediately affected the dissolution rate of target analyte [28]. From the extraction rates of target compounds which were affected by [BMIM]PF_6_ and [HMIM]PF_6_, with the extension of alkyl chain, the enrichment factor of the target compounds was decreased, which indicated that different alkyl chains had an influence on the density of ionic liquids, and water solubility etc. [29]. Therefore, in the following experiments, [BMIM]Br was chosen as the extraction solvent.

#### Selection of type of non-ionic liquid disperser solvent

**Fig. 3:**
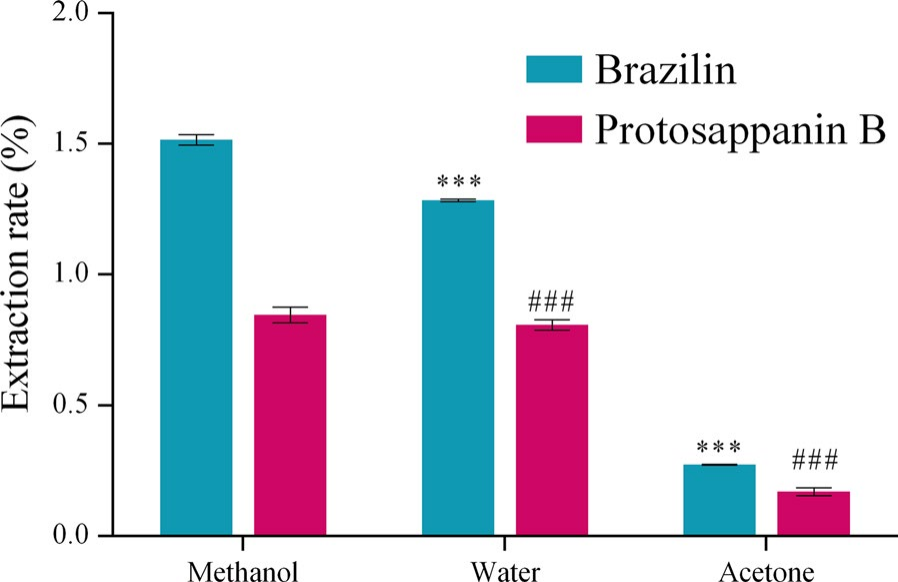
Influence of dispersants on the extraction rate of the compound. Note: compared with methanol (***P ≤ 0.001 or ^###^ *P* ≤ 0.001)

Different dispersants on dispersion ability of [BMIM]Br are different, which have an influence on the extraction rates of target compounds. As it can be seen in Fig. 3, disperser solvent is required to be able to completely dissolve the extraction solvent and easy to dissolve the sample. At the same time, the chromatographic conditions and the determination of analytes need to be well [30]. Methanol, water, acetone, ethanol and acetonitrile were used as the disperser solvents to determine the effect of disperser solvents on extraction efficiencies of two compounds.

Extraction rate was the highest one when methanol was used as the disperser solvent, followed by water. When acetone was applied as the disperser solvent, the extraction rate decreased, as compared with the methanol and a great height miscellaneous peak was appeared in about 5 min. When ethanol and acetonitrile were applied as the disperser solvent, a pair of shoulder peaks appeared in brazilin and protosappanin B corresponding time.

#### Selection of extractant concentration

**Fig. 4:**
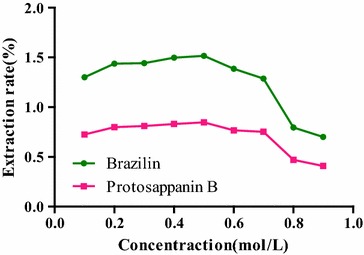
Influence of extractant concentrations on the extraction rate of the compounds

In Fig. 4, with the increase of concentration of [BMIM]Br methanol solution, the extraction rates of two compounds were correspondingly increased. When the concentration reached 0.5 mol/L, extraction rate was the maximum, which were 1.52 and 0.85%, respectively. After this, the extraction rate was reduced the increasing concentration. In view of this, the 0.5 mol/L of [BMIM]Br methanol solution was finally chosen as the extractant in the experiment. Because the extractant viscosity was increased with the increase in extractant concentration, the extraction efficiency was affected, thus, the extraction rate turned to a downward trend after reaching the optimum concentration.

#### Selection of mesh

**Fig. 5:**
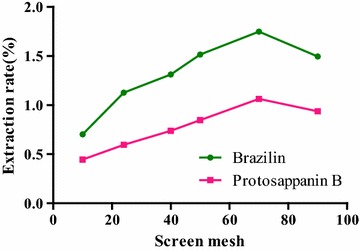
Influence of meshes on the extraction rate of the compounds

The effect of changes in mesh on extraction rate was shown in Fig. 5. With the increase of mesh sieve, the extraction rates of two kinds of compounds were correspondingly increased. The extraction rates of two compounds reached the maximum at 70-mesh. But when the mesh was too small, sample particle started to gather into cluster in ionic liquids, the target compound could not be extracted, the extraction efficiency was affected. Thus, their extraction rates were both increased firstly and then decreases finally.

#### Selection of solid–liquid ratio

**Fig. 6:**
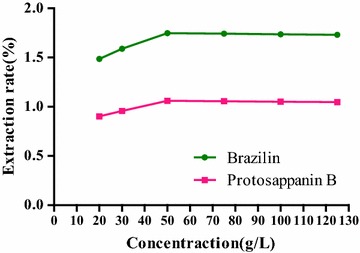
Influence of solid–liquid ratios on the extraction rate of the compounds

The influence of solid–liquid ratios on the extraction rate was shown in Fig. 6. When the quality of medicinal powder was the same, the extraction rate of two kinds of compounds corresponding increased with the increase of the volume of extractant. The extraction rates of brazilin and protosappanin B reached the maximum at the solid–liquid ratio of 1:50. When the solid–liquid ratio continued to increase, the curve tended to a balance. Within certain range of solid–liquid ratio, the dissolution rates of two compounds were increased with the increase in solid–liquid ratio raised. When the ratio continued to increase, the target compound in the medicinal materials were almost extracted completely by ionic liquid, both solubility and dissolution rate reached the maximum.

#### Selection of ultrasonic time

**Fig. 7:**
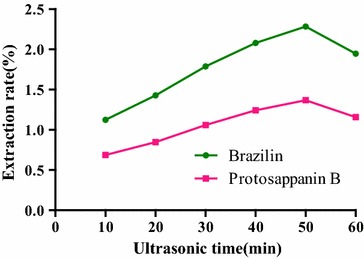
Influence of ultrasonic times on the extraction rate of the compounds

The effect of ultrasonic times on the extraction rate was shown in Fig. 7. Along with the extension of time, extraction rates of brazilin and protosappanin B were increased. The extraction rates of brazilin and protosappanin B reached the maximum after 50 min. When the ultrasonic time continued to increase, the target compound in the sample was destroyed, causing the extraction rate turned to a downward trend, but the peak area of impurity was increased. In view of this, the ultrasonic time of 50 min was the optimal time in the experiment.

#### Selection of ultrasonic temperature

**Fig. 8:**
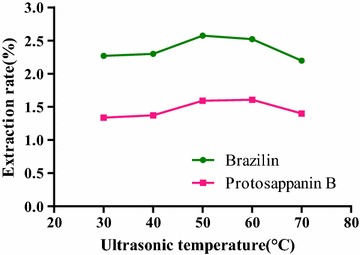
Influence of ultrasonic temperatures on the extraction rate of the compounds

The effect of ultrasonic temperatures on the extraction rate was shown in Fig. 8. Along with increasing of ultrasonic temperature, the extraction rates of two compounds were increased. The extraction rate of brazilin reached the maximum at 50 °C while that of protosappanin B reached the maximum at 60 °C, but was increased only slightly as compared with that at 50 °C. When the ultrasonic temperature continued to increase, effective components in the sample were destroyed, resulting in downward trend of the extraction rates of brazilin and protosappanin B. The extraction rate of brazilin was dropped sharply at 60 °C as compared with that at 50 °C, thus, the ultrasonic temperature of 50 °C was determined as the optimum temperature.

#### Selection of heating time

**Fig. 9:**
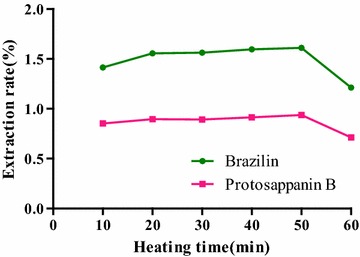
Influence of heating times on the extraction rate of the compounds

The effect of heating times on the extraction rate was shown in Fig. 9. With the extension of time, extraction rates of the two compounds were increased slowly, extraction rates of brazilin and protosappanin B reached the maximum at 50 min. When the heating time continued to increase, the target compound in the sample was destroyed, resulting in the downward trend of the extraction rates of brazilin and protosappanin B. Thus, the heating time of 50 min determined as the optimal time.

#### Selection of heating temperature

**Fig. 10:**
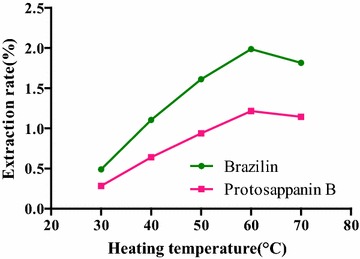
Influence of heating temperatures on the extraction rate of the compounds

The effect of heating temperatures on the extraction rate was shown in Fig. 10. The extraction rates of the two compounds were increased rapidly with increasing the heating temperature. The extraction rate of brazilin and protosappanin B reached the maximum at 60 °C. When the heating temperature continued to increase, effective components in the sample were destroyed, resulting in the downward trend of the extraction rates. Thus, the heating temperature of 60 °C was determined as the optimum temperature.

#### Selection of extraction method

**Fig. 11:**
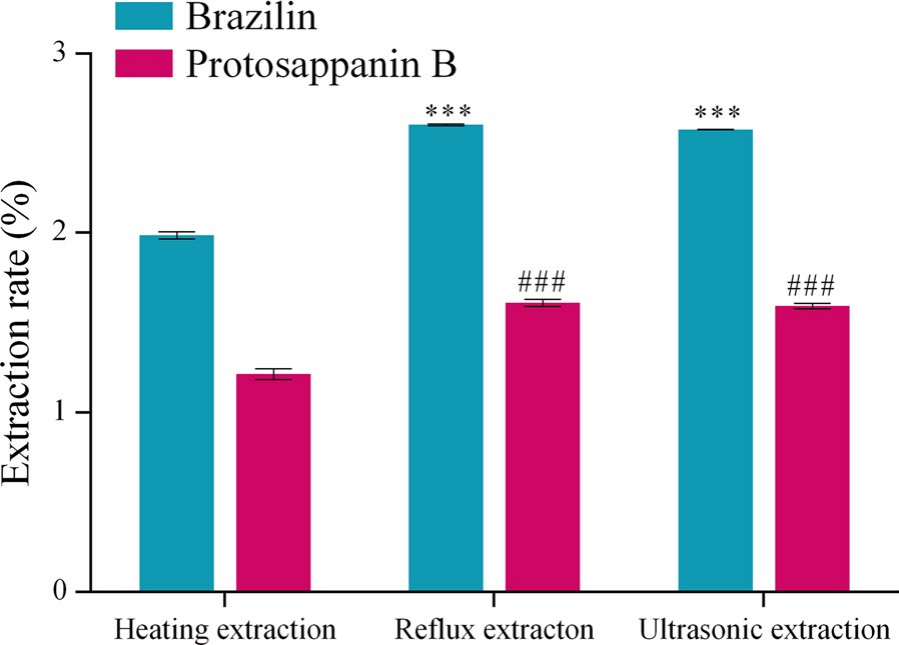
Influence of extraction methods on the extraction rate of the compound. Note: compared with heating extraction (***P ≤ 0.001 or ^###^ *P* ≤ 0.001)

In Fig. 11, the extraction rates of brazilin and protosappanin B were 1.99 and 1.22% with the optimum heating extraction method, respectively. The extraction rates were 2.58 and 1.59% with the optimal ultrasonic extraction method, respectively. The extraction rates were 2.60 and 1.61% with the heating reflux extraction methods under the condition of the same amount of time, respectively. When the heating reflux extraction was adopted to extract the active ingredient, the extraction rates of the two compounds were both higher than that of ultrasonic extraction. However, the heating reflux extraction operation had some defects of assembly. So, the ultrasonic extraction was adopted finally.

#### Selection of centrifugal rotational speed

**Fig. 12:**
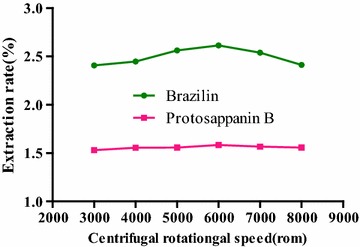
Influence of centrifugal rotational speeds on the extraction rate of the compounds

The effect of the centrifugal rotational speeds on the extraction rate was shown in Fig. 12. With the increase of centrifugal rotational speed, the extraction rates of two compounds were raised. The extraction rates of brazilin and protosappanin B reached the maximum, which were 2.61 and 1.58% at 6000 r min^−1^, respectively. When the influence of the centrifugal rotational speed on the extraction rate was investigated, results showed that the minimal extraction rates were 2.41 and 1.53% at the speed of 3000 r min^−1^, respectively, as compared with the maximum, which were reduced by only 0.2 and 0.05%. In the experiment, centrifugal rotational speeds at 6000 r min^−1^ was chosen as the optimal centrifugal rotational speed finally.

### Evaluation of method performance

#### Precision experiments

The mixed reference substance solution with the injection volume of 10 μL was determined by HPLC, respectively, according to the chromatographic conditions. The relative standard deviations (RSD) were calculated to be 0.29 and 0.30%, respectively.

#### Stability experiments

The test sample solution with the injection volume of 10 μL was determined at 0, 3, 6, 9, 12 and 24 h by HPLC, respectively, according to the chromatographic conditions. RSDs were calculated to be 0.94 and 1.79%, respectively.

#### Repeatability experiments

Six samples were selected on the same time, and 10 μL of these solutions was injected into chromatographic instrument for analysis. The RSDs were calculated to be 1.33 and 1.26%, respectively.

#### Recovery rate experiments

Six samples of *C. sappan* powder (60.00 mg) were divided into two parallel copies and three levels. The standard substances of brazilin and protosappanin B were added whose qualities were 80, 100 and 120% test sample. Each test solutions with the injection volume of 10 μL was determined by HPLC. The recovery rate, the average recovery rate and the RSD were calculated. The average recovery rate of brazilin was 99.33%, the RSD was 1.34%; the average recovery rate of protosappanin B was 98.31%, and the RSD was 1.80%.

### Determined quality of sample

The optimum extraction conditions could be obtained by the test of the influencing factors on extraction efficiency, the peak areas of brazilin and protosappanin B in the methanol extract and the optimum extraction conditions of ionic liquid extract were determined, respectively. The quantities of brazilin and protosappanin B were calculated by the regression equation, the percentages of the corresponding materials in crude drug were finally concluded. In Table 1, the percentages of brazilin and protosappanin B in the ionic liquid extract were higher than those in the methanol extract.

**Table 1:** Percentage of composition of brazilin and protosappanin B in *Caesalpinia sappan* (*n* = 3)

Methanol extractant		[BMIM]Br methanol solution extractant	
Percentage of brazilin (%)	Percentage of protosappanin B (%)	Percentage of brazilin (%)	Percentage of protosappanin B (%)
1.30	0.71	2.61	1.59

#### Antibacterial activities of different extracts

##### The inhibition zone

In Table 2, ionic liquid extract had a strong antibacterial activity against SA, MRSA and *β*-Lactamase producing *S. aureus*. The inhibition zone diameter against MRSA was the largest one, which was 19 mm. Methanol extract also had antibacterial activities against SA, MRSA and *β*-Lactamase producing *S. aureus*. The inhibition zone diameter against MRSA was the largest one, which was 11 mm. The inhibition zone diameter of brazilin against *β*-Lactamase producing *S. aureus* was the largest one, which was 18 mm. However, protosappanin B did not have antibacterial activity. Streptomycin against gram-negative bacteria was valid, but the sample solution and standard solution against gram-negative bacteria were invalid.

**Table 2:** The inhibition zone of extract and standard solutions against SA, MRSA, *β*-Lactamase producing *S.aureus*, EC, ESBLs-EC and PA

Sample	Inhibition zone (mm)						
	Concentration	Bacteria					
		SA	MRSA	*β*-Lactamase producing *S. aureus*	EC	ESBLs-EC	PA
Ionic liquid extract	2.4 mg crude drug/disc	15	19	14	–	–	–
Methanol extract		10	11	8	–	–	–
Brazilin	80 μg/disc	12	12	18	–	–	–
Protosappanin B		–	–	–	–	–	–
Streptomycin	16 μg/disc	18	9	19	19	8	19
Ionic liquid	–	–	–	–	–	–	–
Methanol		–	–	–	–	–	–

Paper diameter is 6 mm; –: no activity; streptomycin: positive control; methanol and ionic liquid: negative control

##### The MIC

In Table 3, ionic liquid extract had lower MIC values against *β*-Lactamase producing *S. aureus*, in which MIC value were only 18.8 mg crude drug/mL. Compared with methanol extract, antimicrobial activities of ionic liquid extract against SA, MRSA and *β*-Lactamase producing *S. aureus* were stronger than those of methanol extract. But the MIC values of ionic liquid extract against SA, MRSA and *β*-Lactamase producing *S. aureus* were higher than those of brazilin and streptomycin.

**Table 3:** The MIC of extract and standard solutions against SA, MRSA and *β*-Lactamase producing *S. aureus*

MIC	Concentration	Bacteria		
		SA	MRSA	*β*-Lactamase producing *S. aureus*
Ionic liquid extract	mg crude drug/mL	37.5	37.5	18.8
Methanol extract		75.0	75.0	150.0
Brazilin	mg/mL	2.5	2.5	2.5
Streptomycin		0.06	0.5	0.03

## Conclusions

The main objectives of this study were to compare thirteen kinds of extraction methods for developing a simple and efficient method for simultaneous determination of brazilin and protosappanin B with HPLC. The results showed that this was the optimal test methods, which was successfully applied to analyze the target analytes. The average recovery rates of brazilin and protosappanin B were 99.33 and 98.31%, respectively. Simultaneous use of ultrasound-assisted, temperature-controlled and ionic-liquid dispersive liquid-phase microextraction reduced the danger of organic solvent. This method is not only environmentally friendly, but also greatly increases the extraction rate of compounds.

## Abbreviations

HPLC: high performance liquid chromatography
[BMIM]PF_6_: 1-butyl-3-methylimidazolium hexafluorophosphate
[BMIM]Br: 1-butyl-3- methylimidazolium bromide
[BMIM]BF_4_: 1-butyl-3-methylimidazolium tetrafluoroborate
[HMIM]PF_6_: 1-hexyl-3-methylimidazolium hexafluorophosphate
